# Genomic characterization of *Salmonella* Uzaramo for human invasive infection

**DOI:** 10.1099/mgen.0.000401

**Published:** 2020-06-26

**Authors:** Xuebin Xu, Yan Chen, Hang Pan, Zaiyuan Pang, Fang Li, Xianqi Peng, Abdelaziz Ed-dra, Yan Li, Min Yue

**Affiliations:** ^1^​ Department of Microbiology Laboratory, Shanghai Municipal Center for Disease Control and Prevention, Shanghai 200336, PR China; ^2^​ Panzhihua Center for Disease Control and Prevention, Panzhihua 617000, PR China; ^3^​ Institute of Veterinary Sciences & Department of Veterinary Medicine, Zhejiang University College of Animal Sciences, Hangzhou 310058, PR China; ^4^​ Panzhihua Municipal Central Hospital, Panzhihua 617000, PR China; ^5^​ Zhejiang Provincial Laboratory of Preventive Veterinary Medicine, Hangzhou 310058, PR China

**Keywords:** *Salmonella *Uzaramo, bloodstream infection, foodborne transmission, whole genome sequencing, sublineage

## Abstract

*
Salmonella* is composed of a wide variety of serovars, causing human self-limited gastrointestinal illnesses or invasive infections. Invasive non-typhoidal *Salmonella* (iNTS) is well documented, with high mortality for children and immunocompromised adults in sub-Saharan Africa and has recently been reported in Southeast Asia. However, iNTS in China remains unknown. In May 2019, a case of invasive infection caused by *Salmonella enterica* serovar Uzaramo (*S*. Uzaramo) was reported for the first time in China. Phylogenomic analysis was performed by genomic sequencing the available contextualized isolates, which separated the two Chinese strains into different sublineages. Both phenotypic and genomic characterization demonstrated that the *S*. Uzaramo isolates showed in general low antimicrobial resistance potential, except one isolated from lake-water in China. Additional comparative genomic analysis and *Caenorhabditis elegans* killing assays suggested a unique combination of virulence factors, including typhoid toxin and *tcf* fimbrial adhesin, which might play a role in the invasive infection. This study highlights that the transparency of global surveillance genomic data could accelerate understanding of virulence and antimicrobial resistance makeup of a previously unknown threat.

## Data Summary

The authors confirm that the data are available under accession numbers SRX7437405 and SRX7437406, and all supporting data or necessary protocols have been provided within the article or through supplementary data file.

*Salmonella *, considered a complex and diverse genus of the family Enterobacteriaceae, comprises two recognized species: *Salmonella enterica* and *Salmonella bongori *. The species *S. enterica* is divided into six subspecies: *enterica*, *salamae*, *arizonae*, *diarizonae*, *houtenae* and *indica* [1]. Based on biochemical and serological characteristics according to the Kauffmann–White Scheme, over 2600 serovars have been identified [2], among which ~1600 serovars belonged to *S. enterica* subspecies *enterica *. However, human infections caused by *Salmonella* are frequently associated with a few serovars, for example serovars Typhi, Typhimurium and Enteritidis (so-called majority serovars). Between 1990 and 2019, in Mainland China, 133 confirmed *Salmonella* serovars were reported in humans [3]. However, the epidemiological significance of a large number of infrequent serovars or minority serovars recovered from humans is usually underappreciated and its clinical importance has been scarcely addressed.

*S. enterica* consists of many non-typhoidal serovars, which are known to cause self-limited gastroenteritis in adults; however, certain serovars can cause severe disease with a greater risk of lethal outcome [4]. Serovar Typhi is the most widely known, causing human typhoid fever, collectively with serovars Paratyphi A, B and C. These four serovars are referred to as Typhoidal *Salmonella *, which are usually human host-restricted and cause invasive infections [4]. The remaining serovars are grouped as non-typhoidal *Salmonella* (NTS), and frequently lead to self-limited diarrhoea, although these organisms may also cause invasive infections, particularly in immunocompromised patients [5–9]. Invasive NTS (iNTS) infections have frequently been documented in sub-Saharan Africa [10], and have recently been reported in Vietnam [11, 12]. Nevertheless, iNTS infection remains largely unknown in China.

NTS is primarily transmitted via commercial food or water. Contaminated raw products are also increasingly considered as an important vehicle for *Salmonella* dissemination, leading to foodborne outbreaks [13, 14]. Reptiles, numerous wildlife animals and, importantly, the environment contaminated by these can also serve as the reservoir for *Salmonella* [15, 16], which are usually found as phylogenetically diverse serovars and isolates [1, 17]. The wildlife and their contaminated environment could reserve for various minority serovars, which are usually different from those recovered from the commercial food-chain. Non-traditional avenues for human infections caused by the infrequent *Salmonella* serovars are usually overlooked; their clinical consequences and human infection causes have rarely been explored. Previously, a case of *Salmonella enterica* serovar Uzaramo (*S*. Uzaramo) was reported to be responsible for bacterial meningitis of a 5-month-old boy, due to the presence of household pythons, but the biological implications for *S*. Uzaramo infection remain obscure [18]. Here, we investigated the first report of *S*. Uzaramo in a bloodstream infection case with invasive syndromes in China. Whole-genome sequencing analysis on the basis of available data indicated that this invasive *S*. Uzaramo strain belongs to one of two defined sublineages. Moreover, the combination of typhoid toxin and *tcf* colonization factor in this strain is probably responsible for the observed human invasive infection.

**Outcome**

There are 2659 identified *Salmonella* serovars, and invasive infections are widely acknowledged for only a few well-known serovars. Current knowledge regarding the causative agents for invasive infections remains restricted to Typhoidal *Salmonella* (caused by serovar Typhi) and a few non-typhoidal *Salmonella* (NTS) serovars, i.e. Typhimurium and Enteritidis. Additionally, most invasive NTS infections have been documented in sub-Saharan Africa and recently in Vietnam. A significant knowledge gap remains for a majority of NTS serovars, and very few studies have reported invasive NTS infections in China. Here, we conducted a clinical investigation, combined with whole genomic sequencing and virulence assays, and demonstrated a strain of *Salmonella* Uzaramo belonging to one of the two identified sublineages was responsible for the observed invasive infection. Furthermore, we detected the typhoid-toxin coding genes, an important virulence factor leading to typhoidal fever, in all Uzaramo isolates, and unique *tcf*, a colonization factor, in the newly defined sublineage Ⅱ in serovar Uzaramo. The discovery of typhoid-toxin-producing *S*. Uzaramo, including those isolated in the UK and USA, poses an emerging public health concern. Our findings advance the field by providing essential knowledge regarding an unusual causative agent of invasive infection, and highlighting the need for genomic data transparency to accelerate the recognition of previously unknown threats.

## Methods

### Case summary

In May 2019, a patient with a clinical history of atrial fibrillation and hypertensive heart disease arrived at a local hospital in Sichuan province. No local or international travel or contact with wildlife or household animals was reported. A meal with rice noodles and raw vegetables outside the hospital was the only significant record for the initial medical history. Acute syndromes of high fever, expiratory dyspnoea, chills and diarrhoea were observed on the next day (Day 2 at 2AM). The patient also complained of headache and abdominal pain. Laboratory investigation indicated an elevated white-blood-cell count (12.97×10^9^/l). Oral berberine and intramuscular injection of gentamicin were given as primary treatment. Two hours later, body temperature had increased to 40 °C, accompanied by watery diarrhoea with tenesmus, nausea and vomiting. Cefoperazone/sulbactam and pantoprazole were administered as new treatment. On Day 3, most syndromes returned to normal, except for nausea, vomiting and watery diarrhoea. On Days 5 and 9, blood and stool samples were collected, respectively. The blood specimen confirmed positive culture for *Salmonella *, but no *Shigella* was detected or isolated from either sample. Sustained treatment with cefotaxime and pazufloxacin was given in the next 3 days and the patient recovered. The patient was diagnosed with infectious diarrhoea, complicated by invasive syndromes.

### Characterization of Chinese *S*. Uzaramo isolates

Four specimens were collected on each occasion (Day 5 and Day 9) for blood and stool samplings, respectively. These specimens were subjected to microbiological analysis. The two stool cultures were negative, while only Day 5 blood samples showed positive colonies on xylose-lysine-deoxycholate agar (XLD agar) plates. The isolated bacteria were subjected to biochemical analysis (VITEK2 COMPACT; bioMérieux) and a PCR test [19].

For comparative purposes, we also investigated additional *S*. Uzaramo isolates in the Chinese Local Surveillance System for *Salmonella* (CLSSS). The CLSSS, which includes the *Salmonella* isolates database for the Centre for Disease Prevention and Control (CDC) over 20 provinces or municipal cities in China, is led by Shanghai Municipal CDC. The CLSSS database has recorded over 50 000 *Salmonella* isolates from human, animal and environmental samples over recent decades. The most recent *S*. Uzaramo isolate U301 was collected from a patient with invasive syndrome in Panzhihua Municipal Central Hospital as illustrated above. The other *S*. Uzaramo isolate U302 was isolated from lake water in Guangxi province (Nanning city), which is geographically close to the Panzhihua city in Sichuan province. All the confirmed *Salmonella* isolates were subjected to a serological agglutination assay according to the Kauffmann–White classification scheme (SSI Diagnostica) [2]. Therefore, we included only two available Uzaramo isolates (U301 and U302) in the following biological investigations.

### Pulsed field gel electrophoresis profiling

In order to compare the genetic relationship between the isolate responsible for the invasive syndrome and other relevant strains, PFGE profiling with restriction enzyme *Xba*I was performed to discriminate these two Chinese *S*. Uzaramo isolates according to the standard protocol as described previously [20]. The Bionumerics v.6.6 software (bioMérieux) was used for clustering analysis and data visualization.

### Antimicrobial susceptibility test

The minimum inhibitory concentration (MIC) of 16 antimicrobial drugs was determined by a broth microdilution assay (Mueller–Hinton broth) as described previously [21–23], with three independent replicates. The MIC range (mg l^−1^) of 16 antimicrobials (Sangon Biotech) used in our assay was as follows: ampicillin (AMP: 0.5–64); amoxicillin and clavulanate potassium (AMC: 0.5–64); gentamycin (GEN: 0.25–32), kanamycin (KAN: 0.5–64), streptomycin (STR: 0.5–64); tetracycline (TET: 0.5–64); ciprofloxacin (CIP: 0.015–8); nalidixic acid (NAL: 0.5–64); chloramphenicol (CHL: 0.5–64); ceftiofur (CF: 0.015–8); cefoxitin (CX: 0.5–64); ceftriaxone (AXO: 0.5–64); trimethoprim and sulfamethoxazole (SXT: 0.06–32); azithromycin (AZI: 0.5–64); ceftaroline (CPT: 0.125–16); sulfisoxazole (SFX: 1–128). The results of the MIC assay were interpreted according to EUCAST Clinical Breakpoint Tables v.9.0 [24]. Otherwise, if not available, the results were analysed with the breakpoints suggested by the U.S. National Antimicrobial Resistance Monitoring System for Enteric Bacteria [25].

### Genomic sequencing and data analysis

Genomic DNA of strains U301 and U302 was extracted from overnight cultures grown at 37 °C in Luria–Bertani broth under 180 r.p.m. shaking conditions by using a TIANamp bacteria DNA kit (Tiangen Biotech). Genomic DNA was quantified using the Qubit Broad Range assay kit (Invitrogen), as per the manufacturer’s instructions. Genome sequencing was performed on an Illumina Nextseq platform using paired-end strategies with a 300-base read length. In addition to the two Chinese strains (U301 and U302), two isolates (AUG147 and 323K), with assembled contigs in FASTA format, from the Enterobase (http://enterobase.warwick.ac.uk/, accessed 31 October 2019) and all available 11 isolates, with raw reads in SRA format, from the NCBI were also included in the comparative analysis.

The raw reads were checked for sequence quality as described previously [20, 26]. Briefly, the quality of sequencing was checked with the FastQC toolkit, and low-quality sequences or joint sequences were removed via trimmomatic [27]. *De novo* assembly and subsequent genomic annotation were performed by using SPAdes v.3.12.0 and Prokka v.1.13, respectively, under the in-house Galaxy platform. The assembled contigs were analysed for plasmids and antimicrobial resistance genes using the CGE PlasmidFinder database (similarity 95 %) [28] and ResFinder database (similarity 90 %) through ABRicate v.0.8 [29]. The Virulence Factors Database (VFDB) was used to screen the potential virulence factors in the examined genomes [30]. Serovar prediction was analysed with two different methods, including SISTR [31] and SeqSero2 [32]. Multilocus sequence typing (MLST) data were analysed by MLST v.2.16.1 [33].

The population structure of 14 available *S*. Uzaramo isolates, including the two newly sequenced Chinese isolates, was investigated, with strain AUG147 as the reference genome and strain S0749 as an outgroup control genome. S0749 was previously described as *S*. Uzaramo in the NCBI but serovar prediction results from SISTR and SeqSero2 both indicated it as serovar Hadar. A total of 57 123 core SNPs were identified in the population of 15 genomes, with a total alignment length of 1 064 234 bases, by Snippy v.4.4.4, as conducted in our previous studies [20, 22]. Core SNPs were used to estimate evolutionary relationships across the *S*. Uzaramo population. A maximum-likelihood phylogenetic tree with 1000 bootstraps was generated using IQ-TREE v.1.6.12 [34] with the best model TVM+F. The phylogenetic tree and associated data were visualized using the Interactive Tree of Life online platform [35].

### *Caenorhabditis elegans* killing assay

To evaluate the virulence potential of two *S*. Uzaramo isolates (U301 and U302), a *Caenorhabditis elegans *killing assay was conducted. Briefly, *C. elegans* SS104 (*glp-4* genotype) was grown at 16 °C, and maintained at 25 °C on nematode growth medium (NGM) plates seeded with *Escherichia coli* OP50. A synchronous population of the worms was required to minimize any variations in the results due to the age difference. For this, gravid worms were washed off using 0.9% NaCl and spun down for 1 min at 1000 r.p.m. Mixed worm bleach liquid (4.5 ml of 0.1 M NaCl, 2 ml of bleach, 1 ml of 5 M NaOH) was added to the pellet and vigorously shaken until the body of worms was disrupted. The egg suspension was then spun down for 1 min at 4000 r.p.m. and washed twice with M9 buffer. Eggs were hatched in M9 buffer in 60 mm Petri dishes agitated at 100 r.p.m. and incubated at 16 °C for 24 h. L1 larvae were then transferred onto NGM plates seeded with *E. coli* OP50, and incubated for growth until they reached the L4 larvae young adult stage.

Bacterial strains (200 µl of 1×10^8^ c.f.u. ml^−1^), including two *S*. Uzaramo strains (U301, U302), and two available control strains (Typhimurium 14028 and SL1344), were cultured on 60 mm NGM plates. After overnight culture, synchronous L4 larvae worms were transferred to NGM plates seeded with test *Salmonella* isolates at 25 °C. Living worms were scored on each of the examined ten days. The assay was performed as three independent experiments with 30 worms per group. The results of the *C. elegans* survival/killing assay were analysed using GraphPad Prism v.6.01. Significant differences were determined using the log-rank (Mantel–Cox) test. *P≤0.05, **P≤0.01 and ***P≤0.001 were considered statistically significant.

## Results

### Clinical investigation

In May 2019, a patient with a clinical history of atrial fibrillation and hypertensive heart disease presented at a hospital in Sichuan province. A takeaway lunch of rice noodles with raw vegetables was recorded on the initial medical history. During the episode, the patient showed invasive syndromes akin to complicated bacteraemia. We collected blood and stool on two occasions (Days 5 and 9) for microbiological analysis. Only on Day 5, the blood specimen showed positive culture on an XLD agar plate. The bacteria were subjected to biochemical analysis (VITEK2 COMPACT; bioMérieux) and PCR diagnostics, which confirmed *Salmonella *. The following serum agglutination assay identified it as serovar Uzaramo (1,6,14,25:z_4_,z_24_:-). No food samples were able to be used for the analysis. Collectively, the bloodstream isolate U301 was suggested as the cause of this episode.

### Characterization of *S*. Uzaramo isolates

**Fig. 1.**
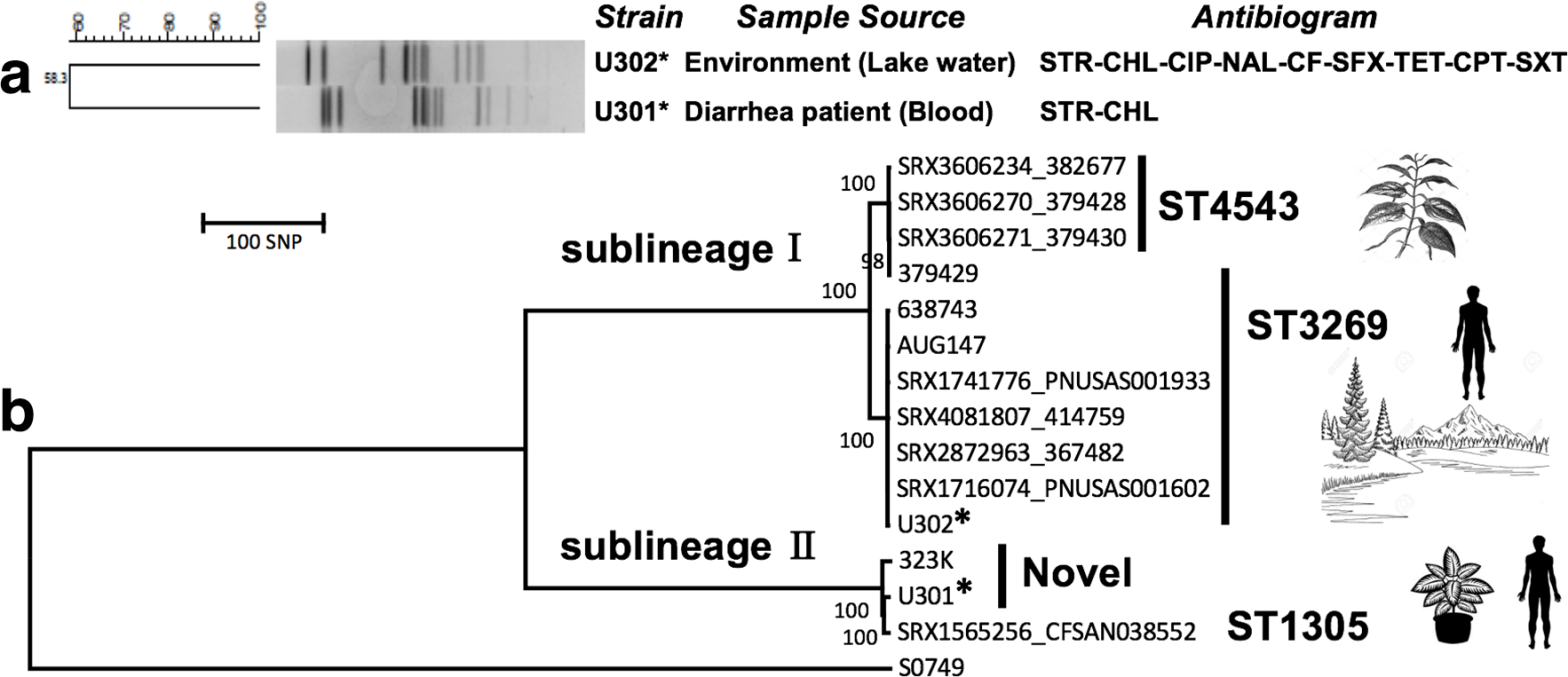
The genomic and genetic diversity of *Salmonella* Uzaramo isolates. (a) PFGE (with XbaI) and antimicrobial resistance profile of the two Chinese isolates. The antimicrobial resistance test was conducted by a broth MIC assay and interpreted according to EUCAST. The detected antimicrobials with resistance were as follows: streptomycin (STR), chloramphenicol (CHL), ciprofloxacin (CIP), nalidixic acid (NAL), ceftiofur (CF), ceftaroline (CPT), tetracycline (TET), sulfisoxazole (SFX) and trimethoprim/sulfamethoxazole (SXT). (b) The phylogenomic relationship among the *S*. Uzaramo isolates. The 14 *S*. Uzaramo isolates were grouped into two sublineages, with ST4543 and ST3269 belonging to Ⅰ, and ST1305 and the novel sequence type (ST) belonging to Ⅱ. The ecological traits for STs or sublineages are labelled with the corresponding symbols. Strains AUG147 and S0749 were used as the reference and outgroup control, respectively. Bar, number of substitutions per nucleotide, corresponding to the evolutionary distance. Asterisks indicate the two Chinese isolates in this study.

For comparative purposes, another *S*. Uzaramo, U302, isolated in 2018 from lake water in Guangxi, was included in the following investigations. Isolate U302 was subjected to the same biochemical and molecular tests, and subsequent serotyping assay to confirm it as *S*. Uzaramo. U301 and U302 were the only two *S*. Uzaramo isolates from a collection of over 50 000 isolates in the CLSSS. Additionally, we performed PFGE profiling and antimicrobial resistance susceptibility tests to compare these two available isolates. Interestingly, these two strains showed a dramatic difference in both PFGE profiles and antimicrobial resistance patterns (Fig. 1a). The *in vitro* broth MIC assay suggested that clinical isolate U301 showed phenotypical resistance to only two antimicrobials (STR and CHL), while the lake-water isolate U302 showed phenotypical resistance for a wide range of antimicrobials (Fig. 1a), including critical antimicrobials, i.e. CIP, CF and CPT.

### Genomic investigation

To further understand the genetic determinants, we performed whole genome sequencing (Illumina Nextseq) for these two available *S*. Uzaramo isolates. To further investigate the diversity and ecological features of *S.* Uzaramo isolates worldwide, we collected genome sequence data from GenBank, SRA archive and Enterobase (accessed 31 October 2019), and we found 12 additional Uzaramo isolates from various sources. On the basis of the assembled genomic data, the two *in silico* serovar prediction methods confirmed these 14 isolates as serovar Uzaramo. Phylogenomic analysis used Snippy v.4.4.4 to obtain an SNP alignment and the phylogenetic tree was built by IQ-TREE v.1.6.12. Two different sublineages were identified, and the two newly sequenced Chinese isolates were separated into either of the two sublineages (Fig. 1b). Importantly, strain U301 was clustered with an isolate from raw macadamia nut (CFSAN038552, ST1305) from the USA in 2015, and a serovar reference strain (323K) of unknown origin. Both U301 and 323K have their own novel ST (Table 1) with independent differences in the nucleotide bases of the *aroC* gene. Together, sublineage Ⅱ has only three isolates, each having a distinct ST. In sublineage Ⅰ, ST4543 and ST3269 were mainly from human and raw food specimens. Interestingly, except for five human isolates (4/5 stool samples), the remaining six isolates, including four from raw betel leaves and two from lake-water, were also associated with the environment, indicating an origin within different environmental niches.

**Table 1.** A list of 14 *Salmonella* Uzaramo strains and one outgroup strain examined in this study ST, sequence type. A dash indicates that data are not available. Tcf indicates an important fimbrial adhesin in *Salmonella *.

Strain	Place	Year	Origin	Age	Note	Accession	Sublineage	ST	Typhoid toxin	Tcf
382677	UK	2017	Food	–	–	SRR6518302	Ⅰ	4543	Yes	No
379430	UK	2017	Food	–	–	SRR6518339	Ⅰ	4543	Yes	No
379428	UK	2017	Food	–	–	SRR6518338	Ⅰ	4543	Yes	No
379429	UK	2017	Food	–	–	RSQJ00000000.1	Ⅰ	3269	Yes	No
638743	UK	2018	Human	–	Faecal	AAHSQF000000000.1	Ⅰ	3269	Yes	No
AUG147	Benin	2008	Water	–	–	SAL_WA2800AA*	Ⅰ	3269	Yes	No
PNUSAS001933	US		Human	40+	Faecal	SRR3474479	Ⅰ	3269	Yes	No
414759	UK	2017	Human	–	Faecal	SRR7163503	Ⅰ	3269	Yes	No
367482	UK	2017	Human	–	Faecal	SRR5633112	Ⅰ	3269	Yes	No
PNUSAS001602	US	2014	Human	50+	Urine	SRR3405887	Ⅰ	3269	Yes	No
U302	China	2018	Water	–	–	SRS5882514	Ⅰ	3269	Yes	No
323K	–	–	–	–	Reference strain	SAL_YA1796AA*	Ⅱ	Novel†	Yes	No
U301	China	2019	Human	50	Blood	SRS5882515	Ⅱ	Novel‡	Yes	Yes
CFSAN038552	US	2015	Raw macadamia nuts		–	SRR3152523	Ⅱ	1305	Yes	Yes
S0749	UK		–	–	Outgroup	ERS106278	Outgroup	770	–	–

*Data from Enterobase: http://enterobase.warwick.ac.uk/.

†A novel ST similar to ST1305 with only one new sequence in *aroC* (closest to *aroC* 351).

‡Another novel ST similar to ST1305 with only one new sequence in *aroC* (closest to *aroC* 351).

Additionally, the results of phenotypic antimicrobial resistance testing could be correlated with the vast majority of antimicrobial resistance determinants in U302 (Table S1, available in the online version of this article). However, U301 has moderate antimicrobial resistance genes or mutations, which were responsible for CHL and STR resistance. Notably, in the remaining 12 isolates that had the genomic sequence data, there were very limited antimicrobial resistance determinants found.

### Evaluation of virulence potential

We further used the genomic sequences to scan the potential virulence factors, by using the VFDB database [30]. Importantly, genes encoding typhoid toxin were detected in all *S*. Uzaramo isolates, while *tcf* fimbriae colonization factor was only found for the isolates from sublineage Ⅱ (Table S1).

**Fig. 2.**
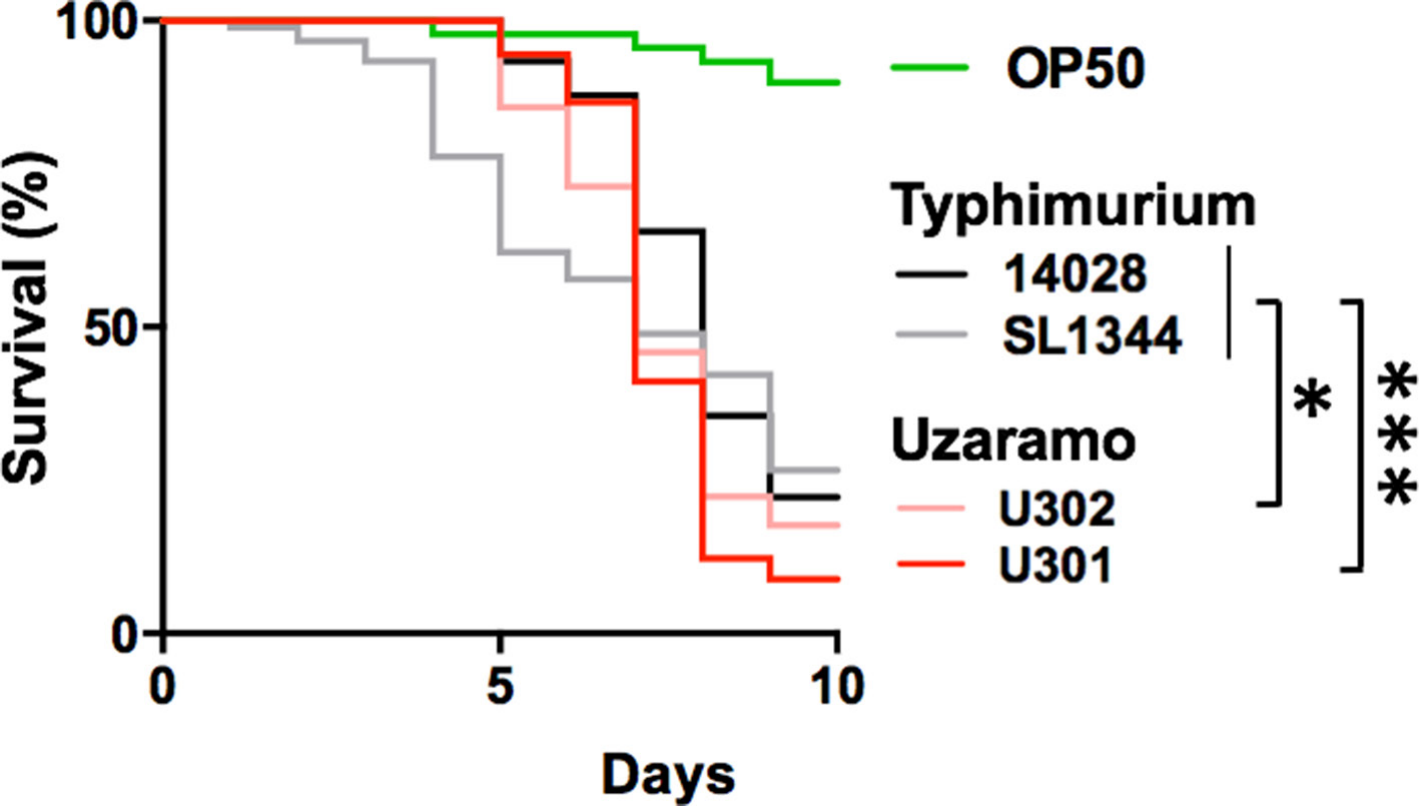
Comparison of three *Salmonella* isolates between two serovars in the *C. elegans* killing assay. *C. elegans* (*n*=30 for each group) was fed with two *Salmonella* Typhimurium strains (14 028, SL1344) and two *Salmonella* Uzaramo strains (U301 from sublineage II, U302 from sublineage I) to evaluate the survival percentage across ten continuous days. Both U301 and U302 had higher killing rates, with statistically significant difference compared with Typhimurium strains 14 028 and SL1344. *Escherichia coli* OP50 was used as the internal control.

In order to evaluate the virulence potential of the two *S*. Uzaramo isolates in an infection model, we used a *C. elegans* killing assay and found that the two *S*. Uzaramo isolates showed a significantly higher killing rate than the two *S*. Typhimurium strains (Fig. 2), which are typical agents causing gastrointestinal diseases. Importantly, strain U301 from sublineage Ⅱ was the best killer among the four isolates, and showed a statistically significant difference (*P*<0.001) in comparison with the two *S*. Typhimurium strains. The carriage of additive particular virulence factors for different sublineages, typhoid toxin only for sublineage Ⅰ and typhoid toxin with *tcf* for sublineage Ⅱ, is consistent with the results of a higher killing rate of *C. elegans*.

## Discussion

*Salmonella* consists of an array of diverse serovars, affecting a wide range of cold- and warm-blooded animals, including humans. Previous studies have reported that fresh edible leaves and cold-blooded animals were reservoirs for *S.* Uzaramo [36]. Only a few cases of human infection caused by *S.* Uzaramo have been reported in the USA and Poland [18, 36], but these lacked clear clinical descriptions. The invasive syndrome observed might also be a result of an immunological condition or medical history of the particular patient, i.e. atrial fibrillation and hypertensive heart disease, in this study. Indeed, clinical investigation of rarely occurring serovars for invasive infections has been hindered by the lack of available information. However, the application of genomic sequencing, in combination with the accumulation of surveillance genomic data, can improve our knowledge about the diversity, possible origin or source, and genetic makeup regarding virulence and antimicrobial resistance of an unusual cause. This information is crucial for improving infectious disease control strategies and subsequent therapeutic treatment when a completely new infectious agent occurs.

In this study, we have reported the first case of *S.* Uzaramo (U301) with invasive syndromes in China, probably due to consumption of raw produce. The invasive *S.* Uzaramo infection isolate was compared with another isolate available in CLSSS collections and showed a dramatic difference in PFGE pattern and antimicrobial resistance profile. Additionally, 12 contextual isolates were included for phylogenomic investigation, confirming the differentiation of *S*. Uzaramo isolates into two different sublineages. Sublineage Ⅰ comprised two STs, ST4543 isolates which are linked to raw produce [37], and ST3269 isolates which have mainly been isolated from human stool samples. U302, a lake-water isolate, was clustered together with ST3269 isolates. However, the meta-data for all these isolates were from a public database without a clear indication of their relationship. It is not known if the raw betel leaves were contaminated via irrigation or washed water. Sublineage Ⅱ contained the invasive isolate U301 identified in this study, in addition to a strain (SRR3152523) linked to raw macadamia nuts, and a reference strain (323K) of unknown origin. Even though the direct relationship between the ingestion of raw produce and the syndromes observed was not proven, we suggest different ecological features in most of the examined *S.* Uzaramo isolates. Moreover, low antimicrobial resistance potential in general, except for lake-water Chinese isolate U302, further supports the probable environmental origin of *S.* Uzaramo [9].

Typhoid toxin was first found in serovar Typhi, and only limited serovars, including at least 47 NTS serovars from multiple serogroup or clades, carry unique toxins [38–41]. Interesting, serovar Panama, which was recently recognized as an extraintestinal iNTS, also carries cytolethal distending toxin (CDT) homology [41, 42]. Here, our comparative genomics approach, for the first time, revealed that all *S*. Uzaramo isolates encoded CDT toxins, mainly by *cdtB*, *pltA* and *pltB* genes. The CDT gene cassette was highly conserved between isolates from serovar Uzaramo and Typhi (data not shown). This toxin is homologous with CDT, suggesting a critical role in the progression of symptoms, including typhoid fever [43]. This may explain the invasive features of *S.* Uzaramo in either young [18] or adult patients, which is also consistent with significant killing in comparison with CDT-negative *S*. Typhimurium. Therefore, CDT-producing bacterial infection may represent an intriguing concern with potential adverse outcomes. Another distinction between the two sublineages of serovar Uzaramo was noted by the presence of *tcf* fimbriae in sublineage Ⅱ isolates, which is considered to play a critical role in virulence of *Salmonella* Typhi. It is suggested to participate in biofilm formation and host-specific colonization [44–46], which may contribute to additive virulence features for invasive U301 in sublineage Ⅱ [47]. Although the *in vitro* biofilm assay shows both of the examined strains, U301 from sublineage Ⅱ and U302 from sublineage Ⅰ, are weak biofilm-producers (data not shown), much work remains to be done to develop an *in vivo* mammal infection model for these strains.

Taken together, the association between invasive syndromes and serovar Uzaramo sublineage Ⅱ has been demonstrated for the first time in this study. In clinics, these less common detected *Salmonella* serovars can probably be misidentified and the actual importance of some clinically relevant serovars may be underestimated. The unique virulence factors carried by minority non-typhoidal serovars may contribute to adverse clinical outcomes, indicating the need for an improved diagnostic approach for those clinically important CDT-carrying serovars, including *S*. Uzaramo. Certain epidemiological information, such as ingestion of raw produce, exposure to environmental parameters or cold-blooded animals, may be of value in the diagnosis of invasive infection caused by *S.* Uzaramo. Furthermore, an enhanced ecological-wide surveillance and monitoring system, with a particular focus on clinically relevant serovars and their corresponding virulence factors, could be of great benefit.

## Supplementary Data

Supplementary material 1
